# Biomechanical Analysis of Defensive Cutting Actions During Game Situations: Six Cases in Collegiate Soccer Competitions

**DOI:** 10.1515/hukin-2015-0029

**Published:** 2015-07-10

**Authors:** Shogo Sasaki, Hideyuki Koga, Tron Krosshaug, Satoshi Kaneko, Toru Fukubayashi

**Affiliations:** 1 Faculty of Health Sciences, Tokyo Ariake University of Medical and Health Sciences, Tokyo, Japan.; 2 Department of Joint Surgery and Sports Medicine, Tokyo Medical and Dental University, Tokyo, Japan.; 3 Oslo Sports Trauma Research Center, Norwegian School of Sport Sciences, Oslo, Norway.; 4 Graduate School of Sport Sciences, Waseda University, Saitama, Japan.; 5 Faculty of Sport Sciences, Waseda University, Saitama, Japan.

**Keywords:** Video analysis, defender, kinematics, center of mass, performance

## Abstract

The strengths of interpersonal dyads formed by the attacker and defender in one-on-one situations are crucial for performance in team ball sports such as soccer. The purpose of this study was to analyze the kinematics of one-on-one defensive movements in soccer competitions, and determine the relationships between lower limb kinematics and the center of mass translation during cutting actions. Six defensive scenes in which a player was responding to an offender’s dribble attack were selected for analysis. To reconstruct the three-dimensional kinematics of the players, we used a photogrammetric model-based image-matching technique. The hip and knee kinematics were calculated from the matched skeleton model. In addition, the center of mass height was expressed as a ratio of each participant’s body height. The relationships between the center of mass height and the kinematics were determined by the Pearson’s product-moment correlation coefficient. The normalized center of mass height at initial contact was correlated with the vertical center of mass displacement (r = 0.832, p = 0.040) and hip flexion angle at initial contact (r = −0.823, p = 0.044). This suggests that the lower center of mass at initial contact is an important factor to reduce the downwards vertical center of mass translation during defensive cutting actions, and that this is executed primarily through hip flexion. It is therefore recommended that players land with an adequately flexed hip at initial contact during one-on-one cutting actions to minimize the vertical center of mass excursion.

## Introduction

Soccer is one of the most popular sports worldwide. In team ball sports such as soccer, the interpersonal dyads formed by the attacker and defender in one-on-one situations appear frequently during competition. In a pilot study, we discovered at least 87 attacker defender dyad situations during nine games in competition that occurred in vital areas. In particular, defensive players have to prevent attackers from breaking through the defensive line while dribbling a ball or making a pass. The ability to quickly change direction is therefore crucial for defensive performance. Unfortunately, the current knowledge of defensive change of direction performance has yet to be determined.

One study calculated the kinematics of offensive players during simulated one-on-one dyad situations with decision-making strategies to evaluate offensive agility performance (Wheeler and Sayers, 2010). Not surprisingly, unanticipated cutting resulted in slower side-stepping compared with pre-planned conditions, unfortunately, joint kinematics were not examined. McLean et al. (2004) investigated the effect of including a static defender on lower limb biomechanics during side-step cutting. The hip and knee angles were found to be more flexed when the defender was included, indicating the importance of keeping a low center of gravity to enable quick changes of direction.

Bradshaw et al. (2011) also measured the sprint time of a pre-planned offensive agility test and suggested that a low center of gravity would be beneficial to maximize the change of direction speed. A likely explanation for these findings is that an upright position before a change of direction will cost time since it is necessary to squat to reach the optimal starting position. However, Bradshaw et al. (2011) did not investigate how the lower center of mass was achieved, for example by flexing the hip or knee.

The essence of all sports is competition. Movements that occur in a laboratory environment will likely be different from movements in real game situations because the playing situation differs substantially, e.g., the laboratory environment does not consider the presence of opponents (Pollard et al., 2007) and teammates (McLean et al., 2004; Wheeler and Sayers, 2010; Kristianslund et al., 2014). Although some studies have investigated the effect of unanticipated maneuvers, these are simple maneuvers where two or three choices must be made. In a real game situation, one does not simply have the choice of cutting 45° left or right, but a complex interaction between teammates and opponents must be assessed before the players’ actions are made. Therefore, analyzing players’ performance during competitions or game situations is necessary to provide the knowledge of how players act in complex and demanding activities.

As a novel biomechanical analysis from video recordings, a Model-Based Image-Matching (MBIM) motion analysis technique has been introduced to investigate human motion from uncalibrated video sequences (Krosshaug and Bahr, 2005). Almost all research using the MBIM method has targeted injury scenes in sports to understand the injury mechanisms, i.e., anterior cruciate ligament ruptures (Krosshaug et al., 2007; Koga et al., 2010; Koga et al., 2011; Bere et al., 2013) and ankle sprains (Fong et al., 2009; Mok et al., 2011; Fong et al., 2012). However, a recent investigation showed the feasibility of the MBIM technique for performance analysis of soccer actions (Sasaki et al., 2013). This application to evaluate sports performance provided a detailed description of joint kinematics and the center of mass translation in on-field sport situations. The previous case report included game and practice situations, and investigated only male players. In the laboratory study by McLean et al. (2004), males used their flexed hip and knee joints more than females when responding to opponent players. There may be a gender difference during on-field sports activities, as shown in a laboratory setting study that was simulated as a one-on-one dyad situation.

The purpose of this study was to describe the kinematics of one-on-one defensive movements in game situations and to determine the relationships between the lower limb kinematics and the center of mass excursion during cutting actions. We hypothesized that the vertical center of mass translation correlated with lower limb kinematics in real games.

## Material and Methods

### Video Collection and Editing

We recorded five soccer games, three male and two female games, in the collegiate division-1 league using at least four digital video cameras surrounding the pitch. The cameras were located around the soccer field (outside the touch and goal lines, which were extensions of the halfway line or penalty area approximately 3–30 m from each line) because multiple perpendicular views were recommended to obtain more accurate results using the MBIM technique (Krosshaug and Bahr, 2005). Basically, an analyst can use any uncalibrated camera from any unspecified location using the MBIM method. Nevertheless, we set general guidelines for the location of cameras (extension of the halfway line or penalty area) in advance, and measured the distance between each camera and the touch or goal lines to achieve more accurate matching. The videos during the male matches were obtained in DV format and standard definition (480i) with a frame rate of 30 Hz (Sony HDR-FX1, Tokyo, Japan; Sony HDR-CX370, Tokyo, Japan; Panasonic NV-GS320, Tokyo, Japan; and Panasonic NV-GS500, Tokyo, Japan), whereas those in female matches were obtained in AVCHD format and high definition (1080i) with a frame rate of 60 Hz (Sony HDR-CX590V, Tokyo, Japan). From these video recordings, six defensive scenes (three males and three females) in which a player was responding to an offender’s dribble attack around the vital area were selected for analysis. Three of the six situations were male players (age 20.7 ± 0.6 yrs; body height 1.75 ± 0.05 m; body mass: 68.5 ± 3.8 kg) and the other three were female players (age 21.3 ± 1.2 yrs; body height 1.63 ± 0.01 m; body mass 58.7 ± 2.3 kg). The players were all participants of university soccer teams in the Japan University Football Association division-1 and the Japan University Woman Football Association division-1. All participants received an explanation of all experimental procedures, and informed consent was obtained before the analysis began.

The video quality was generally good, although fast-moving body parts were slightly blurry. Combining more video clips improves the accuracy of the estimated data (Krosshaug and Bahr, 2005), and previous research also succeeded in motion reconstruction using somewhat blurry images (Koga et al., 2010). In addition, the surrounding images were helpful for accurate matching, even if small parts of the body were less visible. Video recordings were converted into uncompressed AVI or AVCHD sequences before further processing in order to avoid loss of quality. The sequences were converted to the uncompressed tagged image file format (TIFF) using Adobe After Effects (CS5, Adobe Systems Inc., San Jose, California). To synchronize the camera views from the same sequences, manual synchronization was performed using key events in each camera view (e.g., ball touching, initial ground contact, and foot-off). The ethics committee of the Tokyo Ariake University of Medical and Health Sciences approved this research.

### Model-Based Image-Matching Motion Analysis

**Figure 1:**
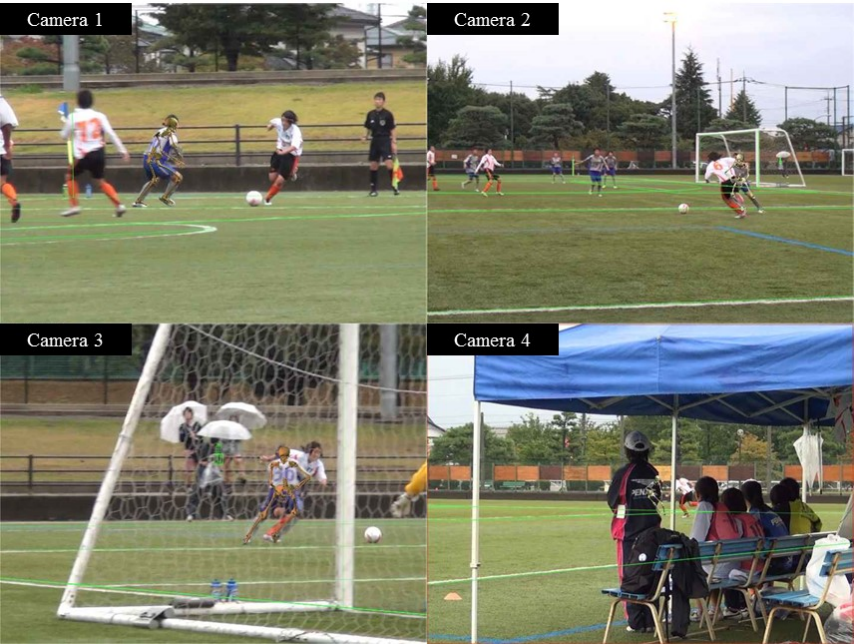
A frame of the matched for case 4 – a female defensive cutting action at the time point of the lowest center of mass height.

To reconstruct the three-dimensional kinematics of the players, we used a photogrammetric MBIM technique (Figure 1). A novel MBIM technique can produce kinematics estimates, the center of mass velocity and acceleration when two or more camera views are available. The accuracy of the MBIM technique was validated in a previous study (Krosshaug and Bahr, 2005). The shapes of the temporal hip and knee joint angles were very similar between the MBIM technique and the marker-based motion analysis using seven infrared cameras. The root mean square hip and knee flexion/extension angle differences were also quite small by the triple camera matching. Details of the MBIM motion analysis for a soccer match were reported previously (Koga et al., 2011; Sasaki et al., 2013). The matching was performed using the three-dimensional animation software Poser 4 and Poser Pro Pack (Curious Labs Inc., Santa Cruz, California). The surroundings were built in the virtual environment according to the real dimensions of the soccer field. The models of the surroundings were manually matched to the background for each frame in every camera view. The skeleton model from Zygote Media Group Inc. (Provo, Utah) was used for player matching. Anthropometric measurements were obtained from five of the six players, and the skeleton model segment dimensions were set based on these measurements. For the last player, the segment dimensions were iteratively adjusted during the matching process until a fixed set of scaling parameters was determined because we could not adjust the participant’s schedule for measuring the length of body segments. Even though no anthropometric measurements were available from previous studies, the skeleton matching was successful (Krosshaug et al., 2007; Koga et al., 2010; Mok et al., 2011). The skeleton matching started with the hip segment and then distally matched with the foot and head segment frame-by-frame. One person performed the matching procedure. To minimize bias resulting from single-operator judgment, another expert gave his opinion on the goodness of the fit. The matching was then adjusted accordingly until a consensus was obtained. The knee and hip joint angles were converted into the joint coordinate system convention of Grood and Suntay (1983). We used Woltring’s generalized cross-validation spline package (Woltring, 1986) with a 7 Hz cutoff to obtain velocity and acceleration estimates for the center of mass translation. The calculations were performed using customized MATLAB® scripts (MathWorks, Natick, Massachusetts) according to previous studies (Krosshaug and Bahr, 2005; Krosshaug et al., 2007; Koga et al., 2010; Koga et al., 2011; Bere et al., 2013; Sasaki et al., 2013).

**Figure 2:**
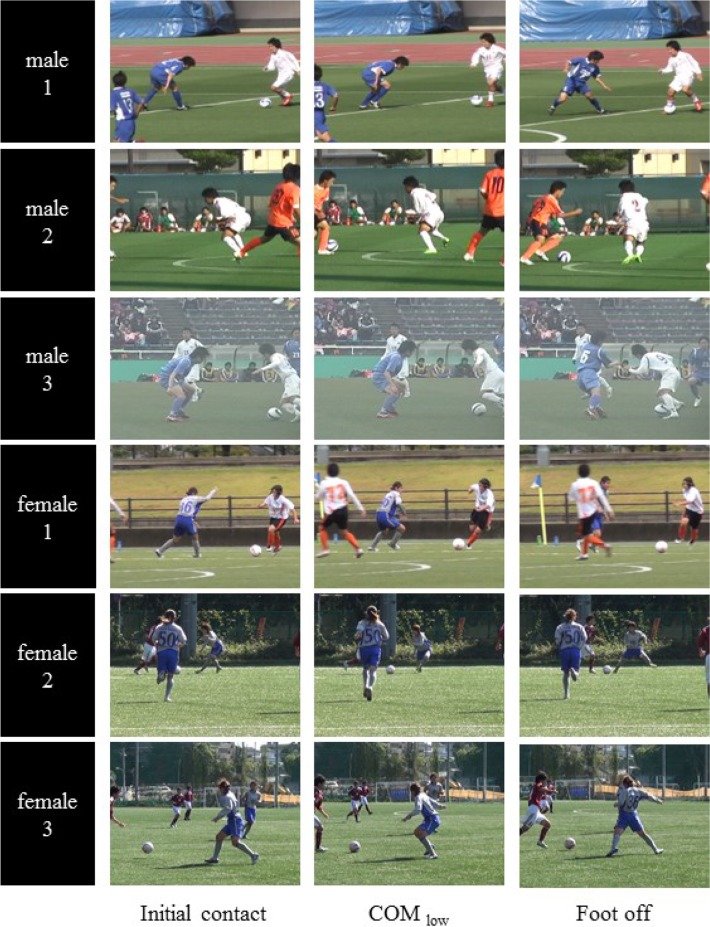
Frame sequences of the six defensive movements at the time point of initial foot contact, the time point of the lowest center of mass height (COM_low_), and the point of foot off.

The center of mass height and the hip and knee kinematics were calculated from the matched skeleton model. In addition, the center of mass height was expressed as a ratio of each participant’s body height in order to normalize the difference in height across the players in reference to a previous study (Shimokochi et al., 2013). Each parameter was analyzed at three different time points: the point of initial foot contact (IC), the point of the lowest center of mass height (COM_low_), and the point of foot off (FO). IC and FO were defined as the first and last frame where the foot contacted the ground to change direction, and were determined visually from the video sequences (Figure 2). Recent video analysis for injured and uninjured players during on-field games also identified feet touching the ground (Boden et al., 2009; Hewett et al., 2009; Sheehan et al., 2012) because devices for calculating ground reaction force do not exist in sports field. They succeeded in finding the frame of the initial ground-foot contact as well as in our study by converting one video to TIFF files and analyzing several consecutive frames. The data collected in the present study did not have a very high sampling rate (30 or 60 Hz); therefore, it was not difficult to see whether a player’s foot had touched the ground. Moreover, we could check each key event more carefully from various angles using multiple camera views, unlike in previous video-based studies.

### Statistical Analysis

All the data were expressed as mean and standard deviation (SD). The relationships between the center of mass height and the kinematics were determined by the Pearson’s product-moment correlation coefficient. All statistical procedures were performed using PASW statistics (ver.18.0 for Windows), and the statistical significance of all tests was set at *p* < 0.05. To confirm the probability of a type II error, post-hoc power analyses were also performed for all statistical analyses and are reported for all significant results.

## Results

**Figure 3:**
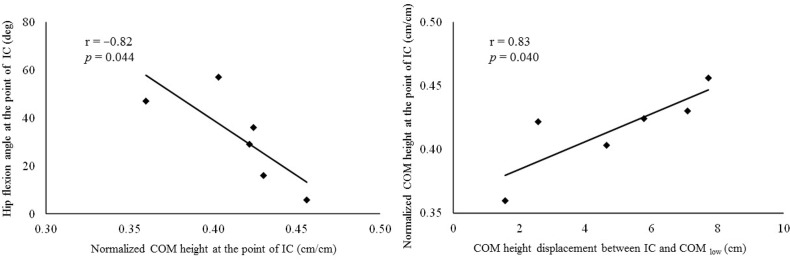
Correlation between the normalized center of mass height at IC and the hip flexion angle at IC (left panel), and correlation between the center of mass height displacement between IC and COM_low_ and the normalized center of mass height at IC (right panel). IC = the time point of initial foot contact, COM_low_ = the time point of the lowest center of mass height

Table 1 shows the descriptive characteristics of the center of mass translation and the hip and knee kinematics variables during soccer competitions. Tables 2 and 3 displayed the Pearson’s product-moment correlation coefficient among the variables. The normalized center of mass height at IC was negatively correlated with the hip flexion angle at IC (r = −0.823 and *p* = 0.044, power .750) (Figure 3). The center of mass height displacement between IC and COM_low_ was positively correlated with the normalized center of mass height at IC (r = 0.832 and *p* = 0.040, power .768) (Figures 3), the angular displacement of the hip joint between IC and COM_low_ (r = 0.870 and *p* = 0.024, power .844), and the knee joint between IC and COM_low_ (r = 0.829 and *p* = 0.041, power .762).

## Discussion

In this study, we described the motion characteristics of cutting actions during several soccer matches, and investigated the relationship between the center of mass translation and the lower limb kinematics during defensive maneuvers. The normalized center of mass height at IC was correlated with the vertical center of mass excursion, and the hip flexion angle at IC. It is therefore likely beneficial to land with an adequately flexed hip at IC during one-on-one cutting actions to minimize the vertical center of mass translation. Although all statistical powers are not sufficient owing to the small sample size in this pilot study, data from real game situations are crucial for our understanding of on-field sports performance. Cohen (1992) proposed that a materially smaller power than .80 would incur too great a risk of type II error. Therefore, our significant results (range of power: .750–.870) would not show a very high error.

Before the statistical analysis, we observed the descriptive characteristics of the center of mass translation and the hip and knee kinematics during soccer competitions. The females tended to display a larger center of mass height excursion and angular changes of the lower limb between IC and COM_low_ compared with the males (Table 1). This may be caused by a lower strength level of female players. It could also indicate a suboptimal movement pattern. Females demonstrated smaller peak hip flexion angles (McLean et al., 2004; Pollard et al., 2007) and knee flexion angles (McLean et al., 2004; Nagano et al., 2011) compared with males during cutting maneuvers in previous laboratory studies. Pollard et al. (2007) found that male athletes were better able to engage the hip extensors in order to control their body during the early deceleration phase of cutting maneuvers. Stearns et al. (2013) also reported that females demonstrated lower hip flexion angles and hip extensor moments compared with males during the deceleration phase for drop jump tasks. With respect to strength, females exhibited decreased isometric strength of the hip joint compared with males (Leetun et al., 2004; Beutler et al., 2009; Stearns et al., 2013). Therefore, it may not be easy for female soccer players to control the vertical center of mass translation and conserve energy during more complicated on-field actions, such as defensive cutting maneuvers.

The normalized center of mass height at IC was correlated with the hip flexion angle at IC (r = −0.823, *p* = 0.044, power .750). Moreover, the center of mass height displacement between IC and COM_low_ was positively correlated with the normalized center of mass height at IC (r = 0.832, *p* = 0.040, power .768), and the angular displacement of the hip and knee joints (r = 0.870, p = 0.024, power .844; r = 0.829, *p* = 0.041, power .762, respectively). These findings suggest that the lower center of mass posture at IC is an important factor to reduce the vertical center of mass excursion during defensive cutting actions, and that this is done through hip flexion. Lowering the body’s center of mass prior to foot contact would be important for developing powerful push-offs and thus fast and quick cutting maneuvers (Shimokochi et al., 2012). Bradshaw et al. (2011) also indicated that a small displacement and low center of mass gravity would be beneficial for offensive agility performance. These findings from laboratory trials support our findings of the field play study. A successful case of a defensive maneuver showed a particularly smaller displacement of the center of mass height compared with an unsuccessful case in previous research (Sasaki et al., 2013). Duarte et al. (2012) suggested that defenders need to quickly adopt postures and movements suitable for actively influencing an opponent’s actions.

Video analysis has limitations, but is perhaps the best approach for obtaining kinematics in real match play. The three-dimensional MBIM technique is non-invasive and can provide three-dimensional joint kinematics. Other studies have used visual inspection analysis (Olsen et al., 2004; Krosshaug et al., 2007) or two-dimensional video analysis (Boden et al., 2009; Hewett et al., 2009; Sheehan et al., 2012) to determine the kinematics during sports activities; however, visual inspection was shown to be inaccurate (Krosshaug et al., 2007). In the future, new sensor technology such as accelerometers may be used to provide estimates of the center of mass variables (Henriksen et al., 2004; Moran and Marshall, 2006).

Some limitations must be kept in mind when interpreting the results of the present study. First, this study is limited to six cases screened for MBIM motion analysis. Since there may be great variation among defensive actions during different match situations, more defensive cases must be analyzed and reported in order to generalize the results of defensive cutting actions in soccer competitions. Second, the estimate using MBIM motion analysis may contain errors. The validation study has shown that the root mean square differences for the hip and knee flexion/extension with three camera views were 2.6° and 7.5°, and multiple and perpendicular views were recommended to obtain more accurate results (Krosshaug and Bahr, 2005). Finally, it is not possible to determine the exact moment of IC, COM_low_, and FO because a relatively low frame rate (30 or 60 Hz) was used in this study. Previous studies that used video analysis discussed the same limitations (Boden et al., 2009; Hewett et al., 2009; Sheehan et al., 2012). Millisecond differences (less than 0.033 s at 30 Hz; less than 0.016 s at 60 Hz) exist in the captured sequences of each of the three points. In addition, we identified the timing of IC and FO visually from various angles. This may lead to millisecond errors because we cannot precisely determine whether a player’s foot has touched the ground. Koga et al. (2011) conducted MBIM analysis using superior-quality videos (high frame rate and high-definition images) from broadcasts and captured detailed motion characteristics. Further studies are required to reduce the errors considered in the present study using high-quality images.

## Conclusions

This study reported defensive cutting actions during soccer competitions using the MBIM technique. The normalized center of mass height at IC was correlated with the vertical center of mass excursion between IC and COM_low_, and the hip flexion angle at IC. It is therefore recommended that players land with an adequately flexed hip at IC during one-on-one cutting actions to minimize the vertical center of mass translation.

## Figures and Tables

**Table 1:** Motion characteristics of defensive cutting actions during football competitions.

		Total (n=6)	Male (n=3)	Female (n=3)
Normalized COM height (cm/cm)	IC	0.42 (0.03)	0.39 (0.03)	0.44 (0.02)
	COM_low_	0.39 (0.02)	0.38 (0.03)	0.39 (0.01)
	FO	0.42 (0.03)	0.42 (0.03)	0.43 (0.03)
Hip flexion angle (deg)	IC	32 (19)	44 (14)	19 (15)
	COM_low_	45 (21)	44 (28)	45 (18)
	FO	2 (16)	0 (9)	4 (23)
Knee flexion angle (deg)	IC	51 (10)	59 (7)	44 (6)
	COM_low_	57 (12)	57 (18)	57 (7)
	FO	24 (8)	25 (10)	23 (8)
COM height displacement (cm)	IC to COM_low_	4.9 (2.5)	2.9 (1.6)	6.9 (1.0)
	COM_low_ to FO	6.5 (2.7)	7.2 (2.3)	5.9 (3.4)
Hip angular displacement (deg)	IC to COM_low_	13 (21)	−1 (15)	26 (19)
	COM_low_ to FO	42 (18)	44 (22)	41 (18)
Knee angular displacement (deg)	IC to COM_low_	6 (12)	−2 (13)	13 (6)
	COM_low_ to FO	33 (18)	33 (25)	34 (12)

IC = the time point of initial foot contact, COM_low_ = the time point of the lowest center of mass height, FO = the point of foot off

**Table 2:** Correlation coefficients between the normalized center of mass (COM) height and the hip and knee flexion angles during cutting actions in football defensive situations

		Normalized COM height		
		IC	COM _low_	FO
Hip flexion angle	IC	−0.823 ^*^	−0.676	−0.275
	COM_low_	−0.027	−0.169	0.347
	FO	−0.299	−0.346	0.086
Knee flexion angle	IC	−0.405	−0.155	0.255
	COM_low_	0.078	−0.114	0.214
	FO	−0.523	−0.455	−0.278

* p < 0.05

C = the time point of initial foot contact, COM_low_ = the time point of the lowest center of mass height, FO = the point of foot off

**Table 3:** Correlation coefficients between the center of mass (COM) height displacement and the normalized COM height, and the hip and knee flexion angular displacement during cutting actions in football defensive situations.

		COM height displacement	
		IC to COM_low_	COM_low_ to FO
Normalized COM height	IC	0.832 ^*^	−0.233
	COM_low_	0.533	−0.183
	FO	0.364	0.438
Hip angular displacement	IC to COM_low_	0.870 ^*^	0.387
	COM_low_ to FO	0.385	0.501
Knee angular displacement	IC to COM_low_	0.829 ^*^	0.096
	COM_low_ to FO	0.504	0.433

* *p* < 0.05

Note: IC = the time point of initial foot contact, COM_low_ = the time point of the lowest center of mass height, FO = the point of foot off
